# Cheap Practitioners

**Published:** 1898-08

**Authors:** 


### ABSTRACTS FROM FOREIGN JOURNALS.

### GERMAN.

UNDER THE DIRECTION OF J. PRESTON HOSKINS,
PRINCETON, N. J.

CHEAP PRACTITIONERS. At the last session of the Lower Austrian Landtag complaints were repeatedly made that the fees demanded by veterinarians were too large in comparison with the value of the animals treated, and it was asserted that the ordinary peasant must therefore have more confidence in an empiric or farrier who was satisfied with a moderate compensation for his cure.

The following incident which really happened, and which is selected from four or five similar ones contained in the *Thierarztliches Centralblatt*, will show that the empiric, or horse-doctor, is not always as cheap as he appears to be.

I was called to visit a sick cow by a cattleowner living outside the city of Vienna. After treating the animal I was asked in regard to the fee. I demanded 50 kreutzers (less than 35 cents) for the visit, and the cattleowner was not a little astonished. "If I had known that, said he, "I would have called you long ago. I always understood that regular veterinarians were very dear." Hereupon he related to me that the year before he had had a fine milch-cow, for which he had paid 120 florins (about $50.00); the animal refused food, and after applying all the common remedies that he knew he called in the empiric, G. The latter decided that the animal had what he called "a nail," and would certainly die, probably very suddenly, and urged that the cow be speedily put to death. After the owner, in order thus to save a part of his loss, had consented to have it slaughtered, the empiric informed him in confidence that it was not advisable to sell the animal to a butcher in the city, for the meat-inspection was very strict there, and the official veterinarian, who did not know very much about the subject anyhow, would have the carcass buried. On the other hand, he knew of a country butcher who could use animals of this kind, and with him there would be nothing to fear. At the request of the owner the empiric finally produced a butcher who offered 25 florins for the animal supposedly sick, and finally bought it for 40 florins. While being driven off the animal began to eat the grass along the road with such avidity that the butcher could hardly get it to move, and the owner begged to be allowed to keep it for at least one day longer. The empiric declared that the cow had only a false appetite and was eating from pain, and so the bargain stood. The empiric received from the owner 1 florin 50 kreutzers for his intervention, and the butcher, having no idea of killing the animal, sold it for 140 florins to another farmer in the neighborhood, where it is to be found to-day as one of his best milch-cows. And still people claim that the cheap and experienced empirics are necessary alongside of the qualified veterinarians.—*Thierarztliches Centralblatt*, March 1, 1898.

